# Patient factors in determining the urgency of cataract referral

**DOI:** 10.22336/rjo.2025.84

**Published:** 2025

**Authors:** Kah Long Aw, Sirindhra Suepiantham, Ashraf Khan

**Affiliations:** 1 Princess Alexandra Eye Pavilion, Edinburgh, United Kingdom; 2 University of Edinburgh, Edinburgh, United Kingdom; Aw KL and Suepiantham S are joint first authors.

**Keywords:** cataract referral, socioeconomic factors, health inequality, nVFQ = Number of symptoms, SIMD = Scottish Index of Multiple Deprivation, VA = Visual acuity

## Abstract

**Objective:**

Patients with cataract experience various symptoms that reduce the quality of life. This study aims to investigate the characteristics of patients who were referred for urgent versus routine cataract surgery.

**Methods:**

A 7-month retrospective study was performed on 140 patients referred by opticians for cataract surgery in the Princess Alexandra Eye Pavilion, UK. Basic demographic information, including the Scottish Index of Multiple Deprivation (SIMD), number of symptoms (nVFQ), visual acuity (VA), urgency of referral by the optometrist, and triage status by the doctor (urgent versus routine), was collected. Sub-group analysis of differences between routine and urgent cases was performed using a two-sample t-test or a Chi-squared test. Logistic regression was used to examine factors associated with an increased odds of urgent presentation. Parameters found to be significant on univariate regression analyses were further investigated using multivariable regression analyses.

**Results:**

Sub-group analysis showed that nVFQ was associated with triage status by doctors (p=0.01) and urgency of referral by optometrists (p<0.0001). VA was not associated with referral urgency or triage status. Univariate logistic regression demonstrated that the nVFQ is associated with triage status by doctors (OR=1.70, CI=1.11-2.60, p=0.014) and urgency of referral by optometrists (OR=2.22, CI=1.44-3.41, p=0.0003). ANOVA testing showed a significant association between SIMD quintile and nVFQ (p=0.036).

**Discussion:**

While visual acuity did not affect prioritisation, patients reporting more symptoms were more likely to receive urgent referrals. Symptom reporting increased with higher socioeconomic status, suggesting reliance on symptoms alone may disadvantage more deprived patients, who already face a higher cataract burden and poorer access to care. These findings align with NICE guidance prioritising lifestyle impact over visual acuity and highlight the need for improved patient education and standardised symptom assessment to promote equitable care.

**Conclusion:**

Our study demonstrated an association between higher cataract symptom reporting, but not visual acuity, with urgency of referral and triage status. Lower socioeconomic deprivation was associated with higher reporting of cataract symptoms. Further research should explore the factors that contribute to the relationship between socioeconomic background and symptom reporting.

## Introduction

Cataract is the leading cause of vision impairment globally, affecting 94 million people [**1**]. Patients with cataract experience reduced quality of life due to decreased visual acuity, increased glare, and loss of colour and contrast sensitivity [**2**]. The only treatment is cataract surgery, whose demand is growing. The United Kingdom Department of Health’s “Action on Cataracts” advises referrals for cataract surgery be based on the following considerations: 1) the patient’s visual acuity, 2) the impact of cataract symptoms on way of life, and 3) the patient’s willingness to have cataract surgery [**3**]. There is variation in the application of these criteria: some clinical commissioning groups (CCGs) in England impose stringent criteria based on visual acuity (VA), others advise the use of scoring systems that incorporate VA and subjective indicators, and others offer no further restrictions beyond the general guidance [**4**]. This variation in referral requirements across areas and hospitals has created a “postcode lottery” for cataract surgery, leaving referrals vulnerable to clinician bias [**5**]. Despite this heterogeneity in cataract surgery criteria, a 2020 study of the national ophthalmology database done by the Royal College of Ophthalmologists concluded that there is limited evidence of inequality in access to primary eye care [**6**].

There is currently no specific national guidance on the timing of cataract surgery (i.e., routine versus urgent). Earlier treatment of cataract with surgery is associated with more positive functional, social, and economic implications and lower rates of surgical complications [**7**]. However, literature on patient factors that affect the urgency of cataract surgery is lacking, and inequitable prioritisation of surgical resources can exacerbate health disparities.

This study, therefore, aimed to investigate the characteristics of patients referred for urgent versus routine cataract surgery. Specifically, we aimed to evaluate the influence of age, sex, socioeconomic background, co-morbidity, visual acuity, and number of symptoms on the timing of cataract surgery.

## Methods

A 7-month retrospective study was conducted from February to August 2022 on 140 patients referred for cataract surgery. The study took place at the Princess Alexandra Eye Pavilion in Edinburgh, UK. Patients deemed suitable for cataract surgery are referred by opticians. Doctors would then review referral letters, and a triage status (urgent versus routine) would be allocated to each patient. The referral letters from electronic patient records were reviewed to gather basic demographic information, including age, sex, postcode, optician postcode, number of symptoms reported by patients, visual acuity, and the urgency of the referral by the optician and the triage status by the doctor. Any missing data was recorded. The referral letters reviewed throughout the study followed the same template, ensuring homogeneity.

During the evaluation of referral letters from patients’ optician appointments, the number of symptoms reported by patients was counted. These mentioned symptoms were then cross-referenced with the list of symptoms provided in the National Eye Institute Visual Functioning Questionnaire-25 (VFQ-25). If a match was found, the patients mentioned these symptoms, which we abbreviated as nVFQ. Interrater variability was minimised by having two investigators screen referral letters for symptoms.

The Scottish Index of Multiple Deprivation (SIMD) 2020v2 was used to assess each patient‘s socioeconomic status by postcode. The SIMD provides a relative measure of deprivation across 6976 small areas, also known as data zones. The SIMD assesses deprivation across seven domains, including current income, employment, health, education, skills and training, geographic access to services, and housing and crime. Each patient’s postcode was assigned a SIMD rank. The SIMD rank ranges from 1 to 6976, 1 being the most deprived. The SIMD quintile represents 20% of a population with a particular level of deprivation. We converted opticians’ postcodes in the same manner.

### Statistical analysis

A subgroup analysis comparing differences between routine and urgent referrals was conducted using a two-sample t-test for continuous variables and a Chi-squared test for categorical variables. Univariate logistic regression was used to examine factors that were associated with increased odds of urgent presentation. Parameters found to be significant in univariate regression analyses were further investigated in multivariable regression analyses to determine whether the relationships persisted. A p-value of <0.05 was considered to be statistically significant. ANOVA analysis was also performed on SIMD quintile and nVFQ. Data were analysed using IBM SPSS Statistics (Version 26).

## Results

Our study comprised data of 140 (81 female) patients who were referred for cataract surgery in PAEP (**Table 1**). We reported no missing demographic information in this study. The mean age of patients was 76.16 (±10.53). The mode of the SIMD quintile was 2. The average VA was 0.62 logMAR. The average VA for patients triaged by doctors as routine and urgent was 0.6 and 0.8, respectively. In comparison, the average VA for routinely and urgently referred patients by opticians was 0.6 and 0.7, respectively.

**Table 1:** Demographics

	Referral triage by a doctor			Referral status by optician		
	Routine	Urgent	*P*-value	Routine	Urgent	*P*-value
**Age, mean**	76.2	75.8	0.86	75.8	77.4	0.47
**AMD, *n***	15	5	0.61	13	7	0.27
**Diabetes, *n***	12	5	0.35	13	4	0.88
**Sex (M, F)**	45,66	14,15	0.45	43,62	16,19	0.62
**VA, logMAR**	0.6	0.8	0.097	0.6	0.7	0.45
**nVFQ**	1.7	2.2	0.01	1.6	2.3	<0.0001
**SIMD Quintile, mean**	2.9	2.8	0.288	2.9	2.9	0.11

Subgroup analysis showed that the number of symptoms reported by patients (nVFQ) was associated with the doctor’s triage status (p=0.01) and the optician’s referral urgency (p<0.0001). Age, AMD, diabetes, sex, VA, and SIMD quintile were not associated with referral status.

### Univariate logistic regression

Univariate logistic regression (**Table 2**) demonstrated that the nVFQ was associated with triage status by doctor (OR=1.70, CI=1.11-2.60, p=0.014) and urgency of referral by optician (OR=2.22, CI=1.44-3.41, p=0.0003). This association remained significant for triage status (OR=1.61, CI=1.02-2.56, p=0.042) and referral urgency (OR=2.07, CI=1.30-3.28, p=0.002) in multivariate logistic regression (**Table 3**). Age, sex, SIMD quintile, and VA were not associated with either triage status or referral urgency.

**Table 2:** Univariate logistic regression for factors associated with doctors’ and opticians’ referral status

	Doctors’ referral status			Opticians’ referral status		
	OR	95% CI	p-value	OR	95% CI	p-value
**Age**	1.00	0.96-1.04	0.86	1.02	0.98-1.06	0.43
**AMD**	0.75	0.25-2.27	0.61	0.27	0.21-1.55	0.27
**Diabetes**	0.58	0.19-1.81	0.35	1.10	0.33-3.61	0.88
**Sex**	0.73	0.602-3.11	0.45	1.21	0.56-2.62	0.62
**VA**	1.82	0.88-3.76	0.11	1.32	0.64-2.71	0.45
**nVFQ**	1.70	1.11-2.60	0.014	2.22	1.44-3.41	0.0003
**SIMD Quintile**	1.18	0.88-1.59	0.26	1.3	0.98-1.72	0.07

**Table 3:** Multivariate logistic regression for factors associated with doctors’ and opticians’ referral status

	Doctor referral status			Doctor referral status		
	OR	95% CI	p-value	OR	95% CI	p-value
**nVFQ**	1.61	1.02-2.56	0.042	2.07	1.30-3.28	0.002

### Anova

We performed an ANOVA test to study the association between SIMD quintile and nVFQ. This showed a significant association [F(4, 135)=2.65, p=0.036].

## Discussion

This study aimed to explore the characteristics of patients referred for cataract surgery in Edinburgh. We found that whilst VA was not associated with prioritisation, patients who reported a higher number of symptoms were more likely to receive an urgent rather than routine outcome from both opticians and doctors. Furthermore, we showed that patients’ SIMD quintile was associated with the number of reported symptoms, with patients in higher quintiles reporting more symptoms.

Our findings provide a valuable extension of an Irish study by Canning et al. (2022), which found that patients were more likely to be listed for cataract surgery if they reported greater detrimental effects of cataract on their lifestyle [**8**]. Furthermore, our results showed that referrals were made in accordance with National Institute for Health and Care Excellence (NICE) guidance (2017 which advises “not to restrict access to cataract surgery based on visual acuity” but to consider the impact of cataract on patients’ lifestyle, despite VA being the most used and perceived as the most suitable indicator of visual function [**9**]. These recommendations were based on a NICE systematic review of published cost-utility analyses, which concluded that setting a VA threshold for cataract surgery in symptomatic patients is not cost-effective due to the progressive nature of cataracts; VA may also underestimate visual disability by failing to account for disabling cataract symptoms such as glare.

Our study found a significant association between SIMD quintile and the number of reported symptoms, with those from more affluent SIMD quintiles reporting more symptoms. This may suggest that prioritising patients for surgery based on reported symptoms alone may be inequitable and contribute to widening healthcare disparities, as evidenced by the relationship between socioeconomic deprivation and increased cataract prevalence, in addition to severity, as observed in several UK and international studies [**10**]. The Singapore Malay Eye study linked lower education and low income to the severity of nuclear cataract and observed a relationship between early cataract intervention and significantly improved quality of life and visual outcomes [**10**]. Our study suggested that individuals from more deprived backgrounds are less likely to report their symptoms; they may experience delayed diagnosis, subsequent delays in appropriate treatment, and thus a prolonged period with an affected quality of life. Mature and hypermature cataracts are also associated with increased rates of surgical complications, ocular co-pathologies, and blindness [**11**]. Discrepancy in symptom reporting between those from deprived and affluent socioeconomic backgrounds could therefore exacerbate existing health outcome disparities and inequities in vision health [**11,12**].

A possible explanation for the relationship between symptom reporting and socioeconomic status is that individuals from less deprived backgrounds may have better health literacy [**13**], leading to greater awareness of cataract symptoms and a higher likelihood of accurately reporting them. Furthermore, those from less deprived socioeconomic backgrounds engage more with eye care services [**14**], which likely enables earlier detection of cataracts and thus greater awareness of symptoms. Evidence for the relationship between socioeconomic status and access to eye health services in the UK itself is weak [**15**]. Therefore, healthcare providers should be aware of these potential disparities and implement targeted outreach and education programs to ensure individuals from all socioeconomic backgrounds are adequately informed about cataract symptoms and encouraged to seek timely care. The widespread use of standardised symptom questionnaires may also ensure that, for those who do present to eye health services, the number of symptoms and their impact on quality of life are accurately and consistently ascertained across all socioeconomic backgrounds.

### Limitations

The findings of our study should be interpreted with consideration of some limitations. Our study was conducted in a specific geographical region, and therefore, caution should be exercised when generalising these findings to other populations. For example, we assumed that all 140 patients included in our study spoke English as their first language and that there was no language barrier hindering the communication of symptoms to opticians. Further studies on health behaviours of different ethnic groups in an English-speaking country would address this limitation. Furthermore, other criteria that may have influenced clinicians’ decisions regarding the urgency of referral, such as medical comorbidities and life expectancy, were not examined in this study.

## Conclusion

Our study showed that patients who reported more symptoms were more likely to be urgently referred by their opticians and triaged as urgent by doctors, resulting in quicker access to cataract surgery. We also uncovered an unexpected association between less socioeconomic deprivation and higher cataract symptom reporting. These findings challenge previous studies, which associate higher socioeconomic deprivation with increased cataract prevalence and severity. While national guidelines recommend that the decision to undergo cataract surgery should centre on reported disease burden, further research should explore the nuanced factors that shape the relationship between socioeconomic background and symptom reporting, to prevent widening healthcare disparities in the context of cataract and broader eye health.
